# O08 - Increased early life Transepidermal Water Loss (TEWL) values can predate atopic dermatitis in asymptomatic infants: results from the BASELINE study

**DOI:** 10.1186/2045-7022-4-S1-O8

**Published:** 2014-03-14

**Authors:** Maeve Kelleher, Audrey Galvin Dunn, Alan Irvine, Hazel Smith, O'B Jonathan Hourihane

**Affiliations:** 1 University College Cork, Cork, Ireland; 2 Trinity College Dublin, Dublin, Ireland

## Background

We sought to ascertain whether a non-invasive measurement of skin barrier function at birth could predict the development of Atopic Dermatitis in asymptomatic infants enrolled in an unselected prospective birth cohort.

## Method

1903 infants were enrolled on the Cork BASELINE Birth Cohort study from July 2009 to Oct 2011. Infants had Transepidermal water loss (TEWL) measured at birth, 2 and 6 months. AD was assessed at 6 and 12 months, using the UK Diagnostic Criteria. Severity was assessed by SCORAD method at 6 months and by both SCORAD and Nottingham Severity Score (NSS) at 12 months.

## Results

The point prevalence of AD in our cohort was 18.7% (299/1597) at 6 months and 15.52% (232/1494) at 12 months. TEWL was lowest at birth, with mean reading 7.32 gwater/m2/hr (±3.33 gwater/m2/hr). It rose between birth and 2 months, mean 10.97 gwater/m2/hr (±7.98 gwater/m2/hr) where it plateaued at 6 months with mean reading 10.71 gwater/m2/hr (±7.10 gwater/m2/hr). A raised TEWL at 2 months was independently predictive of Atopic dermatitis at 12months, but not 6 months when controlling for mode of recruitment, parental atopy and presence of AD.

## Conclusion

Increased Transepidermal water loss is a non invasive signal for skin barrier impairment seen at 2 months in asymptomatic infants prior to clinical appearance of Atopic Dermatitis. This signal is not seen at birth. This finding has implications for the possible prevention of AD if sufficient intervention was put in place to maintain the skin barrier, prior to the appearance of AD.

